# The timeline of epigenetic drug discovery: from reality to dreams

**DOI:** 10.1186/s13148-019-0776-0

**Published:** 2019-12-02

**Authors:** A. Ganesan, Paola B. Arimondo, Marianne G. Rots, Carmen Jeronimo, María Berdasco

**Affiliations:** 1 School of Pharmacy, University of East Anglia, Norwich, NR4 7TJ UK; 2 Epigenetic Chemical Biology, Institut Pasteur, CNRS UMR3523, 28 rue du Docteur Roux, 75724 Paris, France; 3 Epigenetic Editing, Dept. Pathology and Medical Biology, University of Groningen, University Medical Center Groningen, Hanzeplein 1, 9713 GZ Groningen, The Netherlands; 4 Cancer Biology & Epigenetics Group, Portuguese Oncology Institute of Porto (IPO Porto), Porto, Portugal; 5 Department of Pathology and Molecular Immunology, Institute of Biomedical Sciences Abel Salazar (ICBAS), University of Porto, Porto, Portugal; 6 Cancer Epigenetics and Biology Program (PEBC), Bellvitge Biomedical Biomedical Research Institute (IDIBELL), Barcelona, Catalonia Spain; 7 Epigenetic Therapies, Josep Carreras Leukaemia Research Institute (IJC), IJC Building, Campus ICO-Germans Trias i Pujol, Ctra de Can Ruti, Camí de les Escoles s/n 08916 Badalona, Barcelona, Catalonia Spain

**Keywords:** Epigenetics, DNA methylation, Histone modifications, Epidrugs, Therapy

## Abstract

The flexibility of the epigenome has generated an enticing argument to explore its reversion through pharmacological treatments as a strategy to ameliorate disease phenotypes. All three families of epigenetic proteins—readers, writers, and erasers—are druggable targets that can be addressed through small-molecule inhibitors. At present, a few drugs targeting epigenetic enzymes as well as analogues of epigenetic modifications have been introduced into the clinic use (e.g. to treat haematological malignancies), and a wide range of epigenetic-based drugs are undergoing clinical trials. Here, we describe the timeline of epigenetic drug discovery and development beginning with the early design based solely on phenotypic observations to the state-of-the-art rational epigenetic drug discovery using validated targets. Finally, we will highlight some of the major aspects that need further research and discuss the challenges that need to be overcome to implement epigenetic drug discovery into clinical management of human disorders. To turn into reality, researchers from various disciplines (chemists, biologists, clinicians) need to work together to optimise the drug engineering, read-out assays, and clinical trial design.

## Background

Seventy-five years ago, the British biologist Conrad Waddington coined the term *epigenetics* to describe the mechanisms by which an organism stably adapts its phenotype to the environment [1]. Over time, this led to the classical definition (Table 1) of epigenetics as a phenotypic variation that does not originate from an underlying change in the organism’s genotype. Such epigenetic modulation of the genome is self-evident for multicellular eukaryotic organisms. For example, although the cells in our body carry an identical genome, they are clearly able to differentiate and produce higher order tissues and organs distinct from one another. This must be achieved by cell-specific variations in gene expression and, although several definitions of epigenetics are being proposed [2], operationally, we can define epigenetics as the structural adaptation of chromosomal regions so as to register, signal, or perpetuate altered activity states.

**Table 1:** Definitions of epigenetics ranging from classical to the translational

Classical	Alterations in biological phenotype without an underlying change in genotype
Biological	Regulation of eukaryotic gene expression through chromatin remodelling
Chemical	Reversible structural modifications of DNA and histone proteins
Drug discovery	Targeting proteins that introduce, recognise, or remove DNA and histone modifications

At the molecular level, epigenetics involves a highly complex and dynamically reversible set of structural modifications within the nucleic acids and histone proteins that constitute the nucleosome [3]. These chemical alterations are catalysed by enzymes that are often referred to as ‘writers’ and result in the addition to DNA or histones of entities ranging in size from a single methyl group (molecular weight of 15 Da) to proteins such as ubiquitin (molecular weight of 8.5 kDa). Such molecular decorations not only directly influence the affinity between DNA and histone proteins but also recruit partner macromolecules such as non-coding RNAs (ncRNAs) and chromatin remodellers. The binding interactions are controlled through so-called ‘reader’ domains that recognise specific features within the chemically modified nucleic acids and proteins. Finally, to ensure the process is reversible, a series of ‘eraser’ enzymes catalyses the removal of the written information ensuring a dynamic character [3].

Epigenetics is a necessary and essential component of an organism’s normal development and its responsiveness to environmental cues [4]. Nevertheless, it can suffer from dysregulation and there are three major scenarios whereby epigenetics is a significant contributor to the origin and progression of human diseases. Firstly, increasing insights are obtained on disease-associated epigenetic abnormalities (both heritable as well as environmentally induced) [5]. These localised differences in epigenetic states between normal and disease tissues can be exploited as disease biomarkers with diagnosis and/or prognosis potential. Tests to study the DNA methylation of specific genes (e.g. *septin 9*, *vimentin* or BMP3, NDRG4) in non-invasive tissues have been FDA-approved for early colorectal cancer screening programmes [6, 7]. A plethora of studies identifying epigenetic differences in non-tumoural diseases are being conducted; however, they have not yet been developed as commercially available devices and are under clinical trial evaluation or preclinical states. As an example, the diagnostic value of DNA methylation at the BDNF promoter is being tested in clinical trials for the treatment of major depression or autisms [8].

Secondly, the patterns of activity or expression levels of an epigenetic protein can be substantially different in a disease state compared with normal physiology. For example, DNA methyltransferases (DNMTs) are responsible for writing the C5-methylation of CpG dinucleotide sequences [4]. In cancer cells, the pattern of methylation shifts from predominantly non-coding DNA to promoter regions within the genome [4]. Since DNA methylation at promoter regions is generally associated with gene silencing, the consequence is the shutdown of pathways, e.g. DNA repair that would normally help prevent tumour cell proliferation. With respect to disturbed transcription levels, the histone-modifying epigenetic eraser histone deacetylases (HDACs), for example, are found to be highly expressed in many cancers and their increased activity is linked to gene repression [9].

Thirdly, somatic mutations involving epigenetic proteins with resulting gain-of-function or loss-of-function that predispose towards disease have been identified [10]. For example, genetic mutations of the DNA methylation machinery are prominent features of many tumours, especially haematological malignancies (i.e. targeting gain-of-function mutations in DNMT3A in lymphomas are under Phase II clinical trials) [11]. Activating mutations of epigenome-modifying enzymes are really interesting because they open a therapy option based on their inhibition. In this regard, mixed-lineage leukaemia (MLL) is caused by a translocation of chromosome 11. The resulting fusion of the MLL gene promotes recruitment of the lysine methyltransferase DOT1L to methylate histones at pro-leukaemia genes and drive aberrant gene transcription [12]. The targeted inhibition of DOT1L selectively decreases proliferation of cancer cells harbouring the translocation but not the normal cells [13].

Importantly, all three families of epigenetic proteins—readers, writers, and erasers—are druggable targets that can be addressed through small-molecule inhibitors. The discovery and development of epigenetic drugs are extensively described throughout this review. Here, we provide the timeline of epigenetic drug discovery and discuss some of the many challenges and promises still ahead of us.

## The first wave of epigenetic drugs: from phenotypic reality to epigenetic dreams

While rational epigenetic drug discovery using validated targets is now a reality, we should not forget that this enlightened state of affairs is a recent phenomenon. Indeed, drug discovery is ultimately based on the demonstration of efficacy rather than precise knowledge of the molecular target. As we shall see, this is how epigenetic drug discovery evolved and the early (and successful) efforts were based solely on phenotypic observations before an epigenetic connection was realised.

### DNA methyltransferase inhibitors

**Fig. 1:**
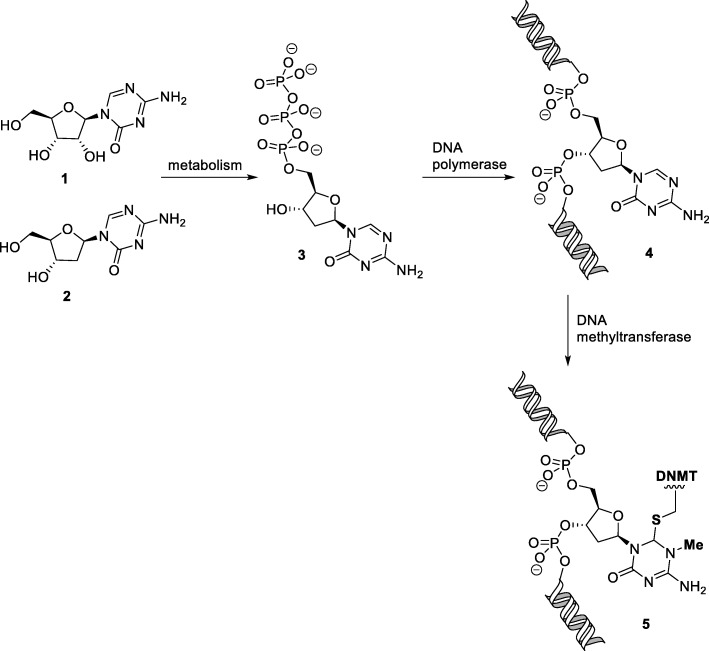
Mechanism of action of azacitidine and decitabine. 5-Azacytidine (compound 1), after metabolic conversion (compound 3) and incorporation into DNA (compound 4), behaves as a suicide substrate for DNA methyltransferases. The enzyme carries out the usual nucleophilic attack by the active site Cys residue and methylation by *S*-adenosyl-l-methionine (SAM) but cannot undergo product release, being trapped instead as the covalent adduct 5, Decitabine (compound 2), has an identical mechanism of action, except that its metabolism to compound 3 does not require a deoxygenation step

In the 1940s and 1950s, the nucleoside building blocks used by Nature to build DNA and RNA became important lead structures for medicinal chemistry. It was believed that structurally related analogues would behave as antimetabolites that interfere with the normal function of the natural metabolites. Some of these compounds inhibited biosynthesis pathways while others were sufficiently similar to be incorporated into nucleic acids and thereby disrupt their function or replication. Relatively subtle modifications of the pyrimidine nucleobase in cytidine have led to drugs such as 5-azacytidine [14] (5-azaC or azacytidine) and 5-aza-2′-deoxycytidine [15] (5-aza-dC or decitabine (Fig. 1). Due to their similarity to cytidine, both compounds are recognised by polymerases and added to the growing chain of nucleic acids. 5-Aza-dC contains the DNA sugar deoxyribose and is incorporated into DNA only, while the ribose sugar in 5-azaC allows incorporation into RNA and DNA after removal of the 3′OH. Compared with the natural nucleic acid building blocks, there is only a single replacement of carbon (C5 in the pyrimidine ring) by nitrogen but this has profound biological consequences. 5-azaC was found to have high antibacterial activity with a MIC_50_ of 250 nM against an *E. coli* strain and toxic in mice with a LD_50_ of 150 mg/kg [16]. The toxicity was primarily manifested in the bone marrow and lymphatic system and suggested the compound might be antileukaemic at a lower dose [16]. Indeed, 5-azaC was cancerostatic at a single dose of 100 mg/kg in a mouse lymphoid leukaemia model. The drug was believed to be a general cytotoxic agent [17] and by 1967 had advanced to clinical trials in Europe. The rapid progress of 5-azaC from bench to bedside led to the American National Cancer Institute (NCI) filing an Investigational New Drug FDA application in 1971. While the clinical trials showed promise in the treatment of acute myeloid leukaemia (AML), the data for other haematological or solid tumours was much less positive [18]. Furthermore, the drug was accompanied by high toxicity and the FDA rejected the application. Subsequently, in a landmark 1980 publication, Jones and Taylor provided the first evidence that cytidine analogues modified at the pyrimidine C-5 position such as 5-azaC inhibit DNA methylation [19]. Importantly, the effect in cells was achieved at lower doses with prolonged exposure whereas higher drug concentrations were detrimental.

The novel epigenetic mechanism of action of 5-azaC and 5-aza-dC (Fig. 1) compared with other clinically used antimetabolites rekindled interest in their therapeutic potential. The most promising application was in the treatment of myelodysplastic syndrome (MDS), a bone marrow disorder with a high risk of progressing to AML that occurs primarily in elderly patients and is characterised by the production of abnormal blood cells. New clinical trials were carried out by the biotech companies Pharmion and MGI Pharma with 5-azaC and 5-aza-dC respectively using lower doses than the earlier investigations [17, 20]. Both drugs successfully received Investigational New Drug (IND) status and in 2004 5-azaC (trade name Vidaza) became the first drug to be approved by the FDA for all five stages of MDS, followed suit by 5-aza-dC (trade name Dacogen) in 2006. The two drugs are currently first-line therapy for MDS when stem cell therapy is not suitable and are additionally used to treat chronic myelomonocytic leukaemia (CMML) and AML [17, 21]. Their success has led to the acquisition of the biotech developers Pharmion by Celgene for $2.9 billion and MGI Pharma by Eisai for $3.9 billion.

### Histone deacetylase inhibitors

**Fig. 2:**
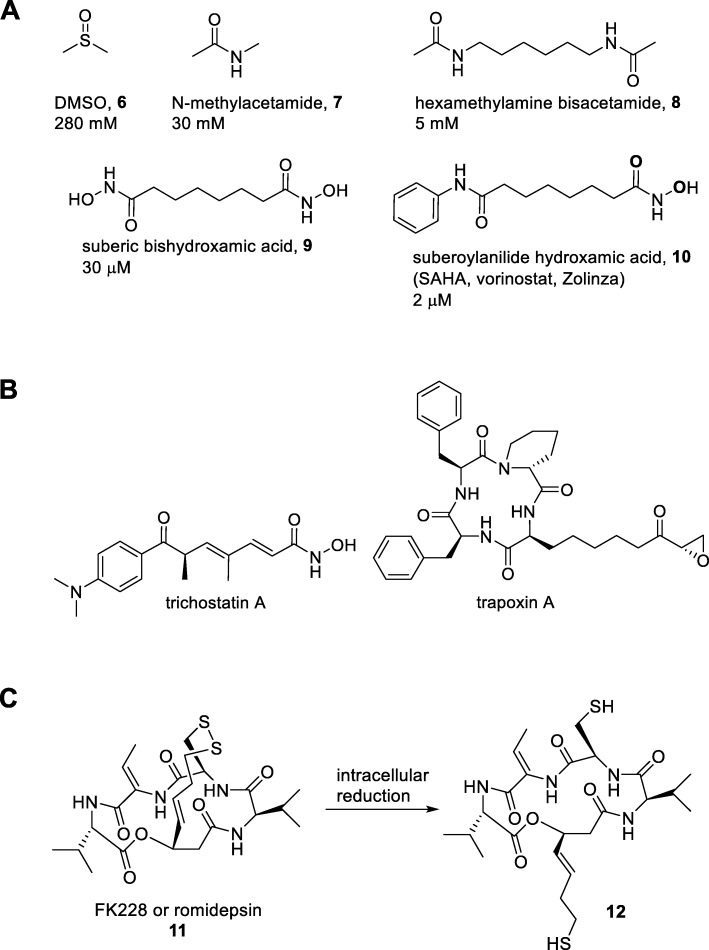
First generation of histone deacetylase inhibitors. **a** Evolution of the HDAC inhibitor vorinostat, with IC50 values indicated for the differentiation of murine erythroleukaemia cells. From the initial observation on DMSO (compound 6), the more potent simple amide (compound 7) was generated, although still at a millimolar level. By employing a classic strategy in medicinal chemistry (so-called ‘multivalency’), compound 8 emerged as a better candidate that entered Phase I/II clinical trials in which partial responses were observed in MDS and AML patients. Attempts to increase the valency through tri- or tetra-amides were unsuccessful, so the strategy moved to considered hydroxamic acids as an amide isostere. The resulting compound 9 was indeed more potent than 8. The original idea behind multivalency was reconsidered and one hydroxamic acid was replaced by an amide to pick up hydrophobic interactions. This process led to the new candidate SAHA (compound 10) which was selected for having the right balance between potency and toxicity. **b** Natural product inhibitors of histone deacetylases (trichostatin A and trapoxin A). **c** Romidepsin and its conversion to the active metabolite. The natural product itself (compound 11) acts as a cell-permeable prodrug that undergoes disulphide bridge reduction within the cellular environment. In the resulting dithiol (compound 12), one of the thiol groups reversibly coordinates to the active site zinc cation in the same way as vorinostat through the latter’s hydroxamic acid.

Vorinostat and romidepsin were the first drugs to be approved that influence epigenetic post-translational modification of histone proteins [22]. Nevertheless, the discovery effort commenced from phenotypic observations without knowing that inhibition of HDACs was involved. The vorinostat story began in 1971 with observations on the ability of dimethyl sulfoxide (DMSO) to cause murine erythroleukaemia cells to differentiate [23]. Ronald Breslow speculated that the mechanism of action involved DMSO binding to its target protein through either metal coordination or hydrogen binding (Fig. 2a). This observation quickly promoted the exploration of a variety of analogues that could make similar interactions [24, 25]. The process led to the development of SAHA (suberoylanilide hydroxamic acid) (Fig. 2a). Thus far, the optimisation process leading from DMSO to SAHA was based solely on phenotypic screening and in vivo studies [26].

Meanwhile, the availability of purified HDACs was enabling identification of the first small-molecule natural products trichostatin A and trapoxin A that specifically inhibit the enzyme (Fig. 2b) [27, 28]. The structural resemblance between trichostatin A and SAHA was noticed, and Breslow soon confirmed that the latter was also a HDAC inhibitor [29]. X-ray crystallographic studies revealed that the hydroxamic acid in SAHA acts as a metal chelator that reversibly binds to the zinc cation in the HDAC active site [29]. SAHA (generic name vorinostat) entered Phase I clinical trials and showed efficacy in a number of cancer types [22]. As further work was not practical in an academic setting, the Breslow set up the biotech company Aton Pharmaceuticals in 2001. The company pursued further clinical trials, with the most promising Phase II results in cutaneous T cell lymphoma (CTCL), a disorder in which malignant cells migrate to the skin and form lesions that can further develop into tumours and undergo metastasis. Aton Pharmaceuticals was acquired by Merck in 2004 for $125 million who completed Phase IIb trials in CTCL. SAHA, now known by the trade name Zolinza, received FDA approval in 2006 and is currently a third-line therapy for CTCL [26]. At present, the clinical applications of vorinostat treatment have been extended to neurological disorders [8], and, interestingly, to the reactivation of persistent viral infection. Actual treatments for HIV-1 patients do not fully eradicate the virus, since it can be latent in CD4+ reservoirs. As the virus latency is mediated, at least in part, by epigenetic mechanisms [30] Phase I/II clinical trials to test the effect of vorinostat treatment in the reactivation of HIV-1 viral latency are being conducted (clinical trial NCT02707900).

Industrial high-throughput screening was the origin of romidepsin, the second HDAC inhibitor to receive FDA approval. At Fujisawa Pharmaceutical, microbial extracts were tested in an assay based on the reversal of phenotype of *ras*-transformed cancer cells and led to the isolation of the active compound FK228 (Fig. 2c) from a strain of the bacterium *Chromobacterium violaceum* [31]. FK228 displayed high activity in tumour models although the mechanism of action was unknown [31]. Later, despite the lack of structural homology with known HDAC inhibitors, this was shown to be FK228’s molecular target [32].

As was the case with vorinostat, Phase I trials with FK228 were most promising for the treatment of CTCL [33]. Later, Gloucester Pharmaceuticals carried out pivotal Phase II trials with FK228, now named romidepsin (trade name Istodax), in CTCL and received FDA approval for this indication in 2009 [34]. The company was acquired by Celgene in 2010 for $340 million and romidepsin received additional approval for a Phase II clinical trial in peripheral T cell lymphoma (PTCL) in 2011 [35]. While vorinostat is a panHDAC inhibitor that inhibits all 11 human isoforms with a submicromolar IC_50_, romidepsin displays selectivity between isoforms with potent nanomolar activity against Class I HDACs (HDAC1, 2, 3, and 8) and a weaker activity against HDAC6.

To summarise, the first four epigenetic drugs were all discovered through phenotypic assays and the road from discovery to approval required long timelines (Table 2). Academic institutions were responsible for the discovery of three of these compounds. The clinical development of all four drugs involved biotech companies who were presumably less risk averse to these experimental targets than big pharma. Once the drugs were approved, however, these companies became acquisition targets for larger organisations. As therapeutics, the DNMT inhibitors 5-azaC and 5-aza-dC have an established and important role as first-line chemotherapy for the treatment of MDS with additional usage in CMML and AML. On the other hand, the HDAC inhibitors vorinostat and romidepsin occupy a much narrower niche despite their initial promise in a variety of tumour types in cell-based and animal models. Their use is restricted to CTCL and PTCL patients when other treatment options have failed [5]. Apart from cancer, the use of SAHA is being explored in neurological disorders, including Huntington’s disease (Clinical Trial NCT00212316) and amyotrophic lateral sclerosis (Clinical Trial NCT00107770).

**Table 2:** Key events in the discovery of the first two waves of epigenetic drugs

	*DNA methyltransferase inhibitors*
1964	Azacitidine and decitabine synthesis
1967	Azacitidine enters clinical trials
1980	Azacitidine and decitabine identified as DNMT inhibitors
1980	Decitabine enters clinical trials
2004	Azacitidine FDA approval
2006	Decitabine FDA approval
	*Histone deacetylase inhibitors*
1971	DMSO reported as differentiation agent
1976	Bisamide precursor to SAHA (vorinostat) reported as differentiation agent
1990	Trichostatin A identified as HDAC inhibitor
1990	Romidepsin reported as a natural product
1996	Vorinostat synthesis
1997	Romidepsin enters clinical trials
1998	Vorinostat and romidepsin identified as HDAC inhibitors
2000	Vorinostat enters clinical trials
2006	Vorinostat FDA approval
2009	Romidepsin FDA approval
2014	Belinostat FDA approval
2015	Panobinostat FDA approval
2015	Chidamide China’s FDA approval

## The second wave of epigenetic drugs: from epigenetic dreams to phenotypic reality

### Second-generation DNMT inhibitors

By the early 1990s, assays for DNMT and HDAC activity were available and potent inhibitors of these enzymes had been identified. With this information in hand, many academic and industrial drug discovery programmes were initiated that aimed to improve upon the early leads. In the case of DNMT inhibitors, the eventually approved 5-azaC and 5-aza-dC have a number of liabilities. The compounds are not selective for a specific isoform of DNMT (i.e. DNMT1 vs. DNMT3s) which might account for some of the side effects. Importantly, 5-azaC is predominantly incorporated into RNA rather than DNA, unlike decitabine. Both drugs have poor bioavailability and are stable only at neutral pH at low temperature, undergoing hydrolysis in either aqueous acid or base. Their half-life is further limited by being accepted as substrates by cytidine deaminase, which hydrolyses the amino group to an inactive 5-azauridine.

**Fig. 3:**
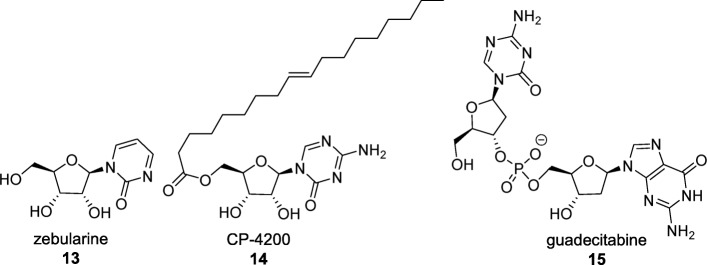
Second-generation DNMT inhibitors. The nucleoside analogue DNMT inhibitors zebularine (compound 13), CP-4200 (compound 14), and guadecitabine (compound 15) have reached clinical development

Several nucleoside analogues that circumvent these shortcomings have reached clinical development although they are not yet in clinical practice (Fig. 3). Zebularine inhibits both cytidine deaminase and DNMTs and has high stability in acidic and neutral pH [36]. CP-4200 is an elaidic acid ester prodrug of 5-azaC with higher bioavailability as it is not solely dependent on nucleoside transporters for drug uptake [37]. SGI-110 (now guadecitabine) is a CpG dinucleotide mimic containing decitabine instead of deoxycytidine; it is not a substrate of cytidine deaminase and hence not subject to this pathway of metabolic degradation [38]. Guadecitabine has been tested in a Phase II clinical trial for the treatment of naïve patients with AML who are not candidates for intensive chemotherapy [38].

In addition, a number of non-nucleoside small-molecule leads have been reported as DNMT inhibitors that are used as preclinical tool compounds against this target [39]. Among these, a typical strategy is to conceive analogues of the *S*-adenosyl-l-methionine (SAM), the methyl donor [40]. These types of derivatives showed interesting results in several cancer models [41–43] and in other human diseases such as parasite infection [44]. The DNMT1 inhibitor hydralazine underwent many clinical trials in the past, both alone and in combinations, and is still in clinical trial (NCT03423810) in polycystic kidney disease patients. In silico models showed that its DNA demethylating activity can be explained by the interaction between its nitrogen atoms with residues Lys162 and Arg240 of the DNA methyltransferase active site [45]. Similarly, procainamide, anti-arrhythmic agent with DNMT1 inhibition activity [46], is under clinical trial against prostate cancer (NCT02103088).

Most recently, bi-substrate analogues have been developed: DNMTs have two substrates, the cofactor SAM, donor of the methyl group, and the cytosine, that will be methylated. By designing analogues of each substrate and linking them together, it is possible to obtain potent inhibitors. This strategy resulted in the most potent chemical tools to inhibit DNMTs to date and they reactivate genes by promoter demethylation in cancer cells [43].

### Second-generation HDAC inhibitors

**Fig. 4:**
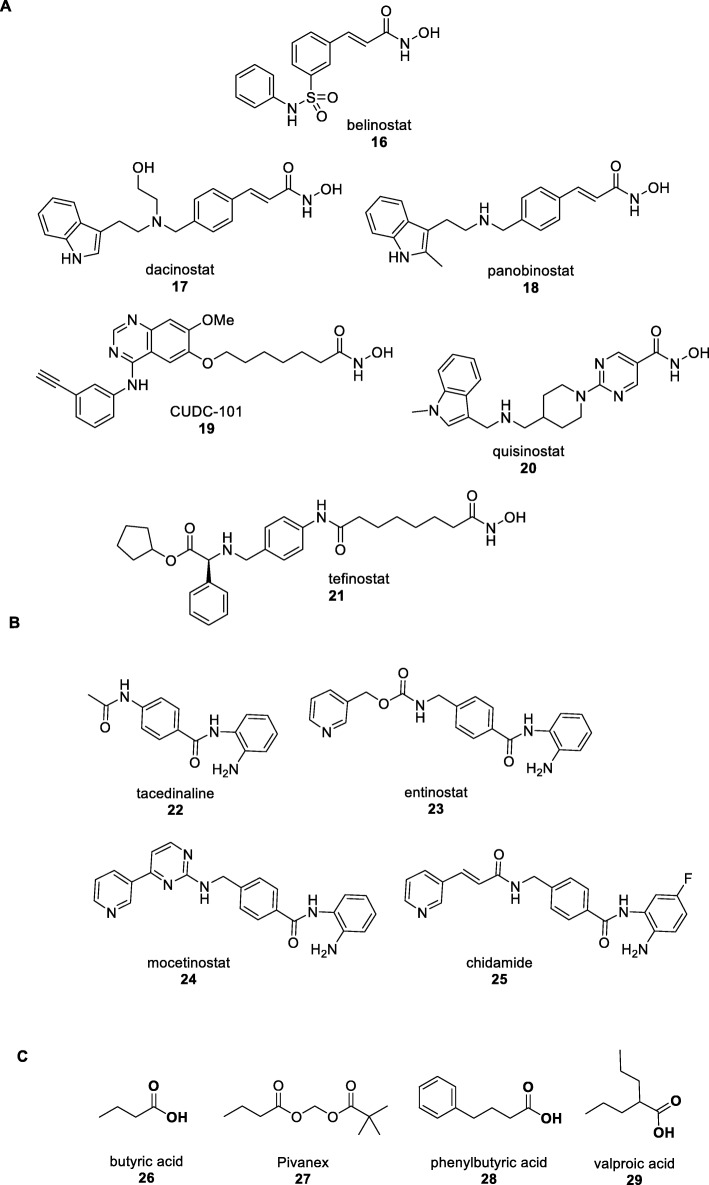
Second-generation HDAC inhibitors. **a** Examples of hydroxamic acid, including belinostat (compound 16), dacinostat (compound 17), panobinostat (compound 18), CUDC-101 (compound 19), quisinostat (compound 20), and tefinostat (compound 21). **b** Benzamide HDAC inhibitors that have reached clinical development, including tacedinaline (compound 22), entinostat (compound 23), mocetinostat (compound 24), and chidamide (compound 25). **c** Carboxylic acid HDAC inhibitors, including butyric acid (compound 26), pivanex (compound 27), phenylbutyric acid (compound 28), and valproic acid (compound 29)

For HDAC inhibitors, the presence of a zinc-binding warhead is key for high-affinity binding to the active site. The hydroxamic acid has proven to be the most successful choice and thousands of synthetic HDAC inhibitors containing this motif have been reported [40]. The early efforts have primarily concentrated on optimising the pharmacokinetic properties of vorinostat which suffers from rapid metabolism and relatively poor bioavailability. The spinout company Prolifix focused on trichostatin A and vorinostat analogues containing more rigid sulfonamide linkers. Ultimately, this would lead to belinostat (Fig. 4a) and the company being acquired by TopoTarget in 2002. In 2010, the biotech Spectrum Pharmaceuticals entered into a co-development agreement with TopoTarget including an upfront payment of $30 million for belinostat. The drug (trade name Beleodaq) received FDA approval in 2014 for the treatment of PTCL [47]. Meanwhile, at Novartis, a reporter assay for increased expression of the p21^Cip1^ gene was screened against their natural product collection and the hits discovered were found to be HDAC inhibitors [48]. A medicinal chemistry effort based on hydroxamic acids delivered the clinical candidate dacinostat with a similar cinnamate linker to belinostat. Due to toxicity concerns, the further development of dacinostat was discontinued in favour of the second-generation candidate panobinostat [49]. In 2015, panobinostat (trade name Farydak) received FDA approval for patients with refractory multiple myeloma that had not responded to previous treatments [50, 51]. Unlike all the other HDAC inhibitors, panobinostat is unique in being approved outside CTCL and PTCL for the more widespread and lucrative indication of multiple myeloma [51]. It is thus far the only HDAC inhibitor to receive approval within the EU. More than ten other hydroxamic acid HDAC inhibitors are currently undergoing clinical trials [52]. Representative examples include CUDC-101 from Curis (a dual mechanism of action HDAC and kinase inhibitor), quisinostat from Janssen (an orally bioavailable compound under investigation for the treatment of solid tumours), and tefinostat from GlaxoSmithKline (designed for targeted delivery to the liver due to reduced efflux after liver specific ester hydrolysis) [52].

Despite the number of hydroxamic acid HDAC inhibitors that have reached clinical development, there are concerns with their pharmacokinetics. Hydroxamic acids are susceptible to clearance by Phase I conjugation and metabolism to toxic species and may have off-target effects due to non-selective metal binding. For these reasons, alternative metal-binding functional groups have been explored although none is completely satisfactory. The aforementioned romidepsin remains the only approved HDAC inhibitor with a thiol zinc-binding group. Although other related natural products and synthetic thiol containing HDAC inhibitors with potent activity have been disclosed, none has progressed to clinical development.

More successful is the use of *ortho*-aminoanilides (benzamides) and the simple example tacedinaline (Fig. 4b) from Pfizer reached Phase II clinical trials before being discontinued (Clinical Trial NCT00005624). At Bayer Schering, the benzamide series produced the candidate entinostat which was later licenced to Syndax who are currently performing Phase III trials in combinatory chemotherapy in breast cancer (Clinical Trial NCT03538171). Concurrently, the biotech MethylGene led to mocetinostat. The company has recently been acquired by Mirati Therapeutics who are investigating mocetinotat in Phase II trials as a single agent and in combination therapy in urothelial cancer or non-small cell lung cancer (Clinical Trials NCT02236195 and NCT02805660). In China, the biotech Chipscreen Biosciences designed a benzamide series with an alkenyl linker from which chidamide was selected as the clinical candidate. In 2015, chidamide (trade name Epidaza) received approval from the Chinese FDA for the treatment of relapsed and refractory PTCL [53]. It is hence the first ‘Made in China’ small-molecule drug for which the entire drug discovery process was carried out within China. As a class, the benzamides display selectivity against class I HDACs with chidamide inhibiting HDACs 1, 2, 3, and the class II HDAC 10 at a nanomolar level [54].

In addition to thiols and benzamides, another zinc-binding motif that has attracted attention for HDAC inhibition is the carboxylic acid. In fact, butyric acid (Fig. 4c) as the sodium salt was the first compound reported to inhibit histone deacetylation as early as 1978 [55]. As butyric acid is rapidly excreted in minutes, the prodrug Pivanex was developed and reached Phase II clinical trials [56]. The HDAC inhibitory activity of butyric acid sparked an interest in other short chain carboxylic acids particularly phenylbutyric acid and valproic acid (VPA), previously approved drugs for treating urea cycle disorders [57] and epilepsy [58] respectively. Although the level of activity is modest (micromolar IC_50_ against the enzyme compared with the nanomolar potency of romidepsin, second-generation hydroxamic acids, and benzamides), their past history and knowledge of human pharmacokinetics have facilitated drug repositioning and their re-examination as anticancer agents in clinical trials [59] or in spinal muscular atrophy (Clinical Trial NCT00227266). While the carboxylic acid family of HDAC inhibitors has not resulted in an approval to date, these compounds are widely used as tool compounds in biological studies.

To summarise, the second wave of epigenetic small-molecule modulators involved analogue-based drug discovery. Taking advantage of the known leads for DNMT and HDAC inhibition, drug discovery projects in biotech and big pharma have culminated in more than 20 clinical candidates. At the moment, this has yielded three approvals for HDAC inhibitors in belinostat, panobinostat, and chidamide. A particularly exciting development was the potential to widen the applications of HDAC inhibitors by the evaluation of second-generation compounds in non-haematological cancers. As single agents, however, these molecules disclosed limited effectiveness. Nonetheless, the combination of HDACi and DNMTi was demonstrated to be beneficial in patients harbouring solid tumours, namely, advanced breast cancer and metastatic lung cancer [60, 61]. Furthermore, the usage of epigenetic therapy in combination with chemotherapeutics of a non-epigenetic nature had been studied in early phase clinical trials for colorectal, cervical, ovarian, and pancreatic cancer [62]. Currently, the combination of epidrugs and immunotherapy is also disclosing promising results in cancer patients’ treatment, including those with colorectal, non-small cell lung, and kidney cancers [62, 63]. Apart from cancer, and as previously explained for vorinostat, the use of panobinostat, chidamide, and romidepsin are under Phase I/II clinical trials for the reactivation of latent HIV-1 latency (Clinical Trials NCT01680094; NCT02513901 and NCT01933594).

## The third wave of epigenetic drug discovery: new dreams, new realities

Since the last decade, the concept of epigenetic therapy has become a reality with the approval of seven small-molecule drugs that inhibit DNMTs and HDACs (Table 2). These success stories have validated the hypothesis that it is possible to regulate an epigenetic process for the treatment of disease and that a therapeutic window can be achieved clinically for this new class of drugs. The race is now on to extend these discoveries to other epigenetic writer and eraser targets and even epigenetic readers are targets for small-molecule inhibition. For three of these in particular, as described below, significant progress has been made and compounds are currently in clinical development.

### Histone methyltransferase inhibitors

**Fig. 5:**
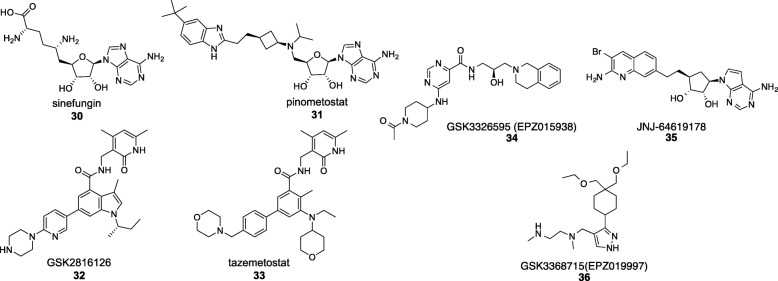
Examples of KMT inhibitors including the clinical candidates sinefungin (compound 30), pinometostat (compound 31), GSK2816126 (compound 32), tazemetostat (compound 33), compound GSK3326595 (compound 34), JNJ-64619178 (compound 35), and GSK3368715 (compound 36)

Lysine histone methyltransferases (KMTs) are the enzymes that post-translationally add one to three methyl groups to lysine residues in proteins. Whereas DNA cytosine methylation and histone deacetylation have a repressive effect, lysine methylation can either activate or silence gene transcription depending on the specific lysine residue involved [64]. The human KMTs are nearly 100 in number and use SAM as the methyl donor. The natural product sinefungin (Fig. 5) reversibly competes with SAM for its binding site and therefore is a non-selective inhibitor of all SAM utilising enzymes [65]. The breakthrough in KMT drug discovery has hinged on the ability to design potent SAM-mimetics that are selective by taking advantage of differences in the cofactor binding pocket [65]. Epizyme’s pinometostat, a highly selective DOT1L inhibitor, was the first KMT inhibitor to enter Phase I clinical trials for the treatment of leukaemia [66]. Then, GlaxoSmithKline and Epizyme have independently employed a pyridone scaffold for the selective EZH2 inhibitors GSK2816126 (NCT02082977) and tazemetostat (NCT01897571), respectively, that are undergoing Phase II trials for B cell lymphoma [67]. Both DOT1L and EZH2 inhibitors have exciting prospects for personalised medicine [64, 68].

Among the arginine methyltransferase inhibitors, the first to enter clinical trials is GSK3326595 (formerly EPZ015938) directed against PRMT5 [69]. PRMT5 overexpression is observed in cell lines and primary patient samples derived from a number of cancers, and clinical trials have started in 2016 in non-Hodgkin lymphoma and solid tumours [69]. In addition, PRMT5 inhibitor JNJ-64619178 (Clinical Trial NCT03573310) as well as PRMT1 inhibitor GSK3368715 (formerly EPZ019997) (Clinical Trial NCT03666988) have now entered clinical trials.

### Lysine demethylase inhibitors

**Fig. 6:**
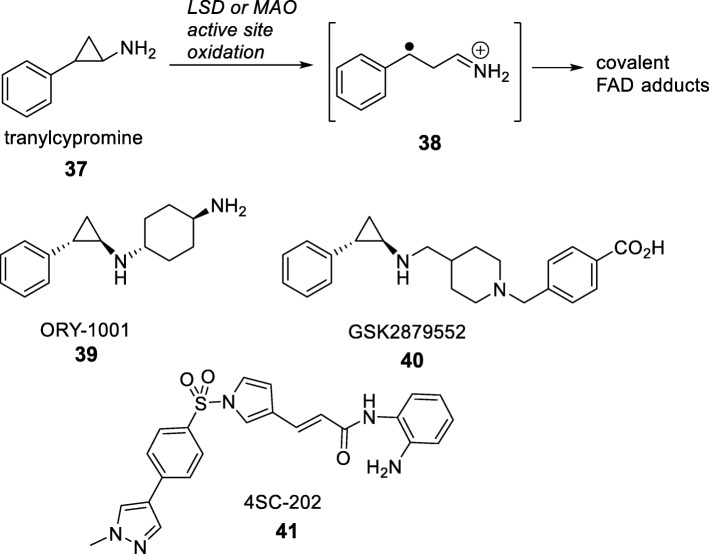
The mechanism of action of tranylcypromine and tranylcypromine analogues in clinical trials as LSD1 inhibitors. The monoamine oxidase inhibitor tranylcypromine (compound 37) binds as a suicide substrate in the MAO or LSD active site and then undergoes strain-induced ring opening to give a reactive radical cation (compound 38). This reactive intermediate covalently adds to the FAD cofactor thereby irreversibly inhibiting the enzyme. Tranylcypromine analogues are being developed to improve inhibition, including ORY-1001 (compound 39), GSK2879552 (compound 40), and 4SC-202 (compound 41)

Lysine methylation is reversed by the lysine demethylase enzymes (KDMs) that are subdivided into two families based on their catalytic mechanism [70]. The larger Jumonji C (JmjC) demethylases comprise about twenty human enzymes classified as KDM2-7. These are part of the 2-oxoglutarate and iron (II)-dependent dioxygenase family and are capable of demethylating mono-, di-, and trimethylated lysine residues. Although there are interesting links between JmjC demethylases and human disease, at present, inhibitors of the enzymes are at the stage of chemical probe development [70]. The second family KDM1 contains the enzyme lysine-specific demethylase that exists as two isoforms LSD1 and LSD2 (also known as KDM1A and KDM1B), which are mono- and dimethyl-lysine demethylases. The LSD enzymes are homologous to monoamine oxidases (MAOs) in their mechanism and similarly use FAD as the cofactor to effect oxidative cleavage of the methyl group. LSD inhibitor design has greatly benefited from this homology as old MAO inhibitor drugs have been repurposed as demethylase inhibitors [70]. The most successful example is tranylcypromine (Fig. 6), an approved MAO inhibitor initially used as an antidepressant [71]. In AML that is resistant to chemotherapy with all-trans retinoic acid (ATRA), LSD1 inhibition by tranylcypromine restores sensitivity to ATRA [72]. As a result, combination therapy with the two drugs is being evaluated in clinical trials (Clinical Trial NCT02273102). Since tranylcypromine is a modest LSD1 inhibitor (IC_50_ ~ 20 μM), many analogues have been reported with a view to improving its activity and reducing off-target effects against other FAD-containing enzymes. Among these second-generation compounds, Oryzon and GlaxoSmithKline have disclosed the structures of their respective clinical candidates ORY-1001 and GSK2879552 in Phase I/II trials [73]. In AML, LSD1’s function as a H3K4 demethylase leads to the maintenance of stemness hence supporting the idea of therapeutic intervention through this target [74].

In the quest of new inhibitors scaffolds, of note is the design and selection of cyclic peptides to target KDMs with high selectivity (such as CP2 for KDM4A-C) [75].

### Bromodomains

**Fig. 7:**
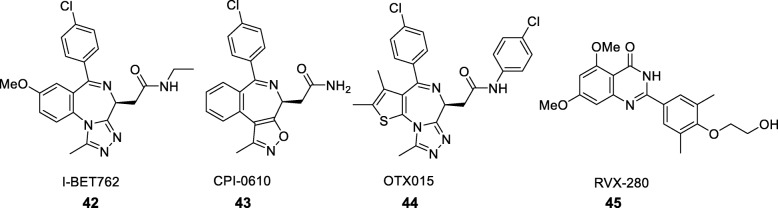
Examples of bromodomain ligands in clinical development, including I-BET762 (compound 42), CPI-0610 (compound 43), OTX015 (compound 44), and RVX-280 (compound 45)

The drug discovery targets discussed above are either writers (DNMTs and KMTs that methylate DNA and histones respectively) or erasers (HDACs and KDMs that deacetylate and demethylate histones respectively). The first epigenetic readers to have a significant impact on drug discovery are the bromodomains [76]. This family of protein recognition domains for acetyl-lysine residues consists of 61 human members. The acetyl-lysine binding region is hydrophobic in nature and can be occupied by small-molecule ligands. Within the bromodomains, the BET (bromo and extra terminal) family comprising BRD2, BRD3, BRD4, and BRDT has been a predominant focus for drug discovery [76] because of their implication in cancer. As an example, BRD4 promotes the expression of oncogenes, while the translocation of BRD3 or BRD4 to the NUT oncogene leads to its constitutive activity and is the cause of NUT midline carcinoma (NMC) [77]. The first small-molecule ligands for bromodomains were discovered serendipitously in pharmaceutical companies through cell-based assays without knowledge of their mechanism of action. At GlaxoSmithKline, for example, an assay for upregulation of ApoA1 transcription identified a series of hits that were then shown by proteomic studies to be potent ligands for the BET bromodomains [78]. Further optimisation produced I-BET762 (Fig. 7), a nanomolar BET ligand that is in Phase I/II trials for NMC and haematological cancers [79]. Due to their implication in multiple biological pathways, BET inhibitors are also being explored in other diseases, including diabetes or reactivation of latent HIV-1 in human cells [79]. Parallel investigations at other pharmaceutical companies with the benzodiazepine scaffold have led to clinical candidates including Constellation’s CPI-0610 and OncoEthix’s OTX015 [80]. While these compounds are being investigated in cancer, Resverlogix’s RVX-208 was discovered through phenotypic screening in an atherosclerosis programme [81]. The compound has recently completed Phase IIb trials that demonstrated increased levels of ApoA1 and high-density lipoprotein (HDL) in patients (Clinical Trial NCT02586155).

To summarise, the third wave of epigenetic drug discovery has witnessed rapid progress with three new targets: the KMTs, KDMs, and bromodomains. The speed from bench to bedside has been breath-taking given that the bromodomain was identified as an acetyl-lysine binding unit only in 1999, EZH2 as the H3K27 methylase in 2002 and LSD1 as a KDM in 2004. In just over a decade, multiple small-molecules for these targets have advanced to clinical trials and it is highly likely that one or more will receive clinical approval in the near future. Although the process of drug discovery has followed the modern paradigm of target validation followed by sequential in vitro and in vivo proof of concept, the role of phenotypic observation should not be forgotten. The bromodomain efforts were accelerated through reporter-based phenotypic assays that led to the discovery of high-affinity ligands whose mechanism of action was only elucidated later. For LSD1, the most promising lead has been tranylcypromine, an old drug with a 50-year history of use as an antidepressant through a non-epigenetic target.

## The fourth wave of epigenetic drug discovery: crystal ball gazing

**Fig. 8:**
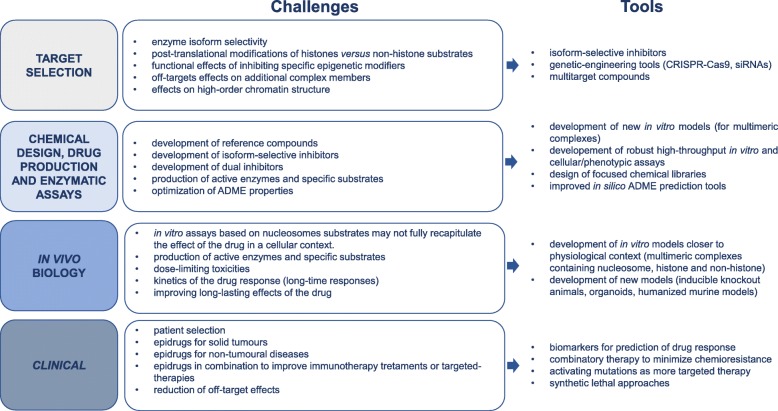
Challenges and opportunities for epigenetic drug discovery

The developments in epigenetic drug discovery [64, 82, 83], together with the growing insights in the importance of epigenetic mutations in diseases [5, 84–86] and consequent biomarker discovery [86], definitely marks epigenetics as an exciting field of research with promising clinical implications. Indeed, the speed of developments in drug design indicates the huge demand for this novel avenue to be fully exploited in order to combat diseases. In this section, we discuss various challenges and opportunities for the implementation on epigenetic drug in clinical use, including aspects as target selection, chemistry-associated concerns, in vivo biology, or clinical considerations (Fig. 8).

Selection of the most appropriate druggable target with functional impact on the disease is undoubtedly a high relevant consideration. Next to rational chemical medicinal engineering approaches, biological approaches (protein complexes or cells) are essential to identify the functional effects of inhibiting particular enzymes or even isoforms [87, 88]. The EU H2020 COST Action consortium CM1406 (www.EpiChemBio.eu) brought together scientists from the different fields (chemistry, biology, medicine) to address such complex issues. The recently introduced CRISPR/Cas approach has raised exponential interest as a tool to correct genetic mutations but also offers powerful possibilities to inactivate any given gene [89]. Moreover, if loss-of-function is caused by epigenetic silencing of expression, CRISPR/Cas can be repurposed to target effectors to that genomic locus to re-express the silenced gene. This latter application of CRISPR/Cas is achieved by mutating the nuclease activity of the Cas protein (resulting in a dead Cas (dCas)) [89]. dCas is still being recruited to the genomic site of interest via single guide RNAs, and dCas can thus be used to shuttle any fused protein to this location. If the catalytic domain of an epigenetic enzyme is fused to dCas, the system will result in rewriting of the epigenetic signature at this particular location (epigenetic editing) [89]. Furthermore, for non-cancerous diseases where cell death is not the final goal, sustainability of the effect is important and can be achieved using epigenetic editing. Interestingly, sustained re-expression of epigenetically silenced genes has been shown for various genes, including tumour suppressor genes [90].

Although an epigenetic target has been identified for a specific disease, several challenges need to be overcome for a successful epigenetic drug design, including enzyme isoform selectivity, the selectivity for histone’s lysines and arginines vs. non-histone substrates, the design of dual inhibitors, how to use the epigenetic drugs in combinations, and the fact that epigenetic enzymes often work in multimeric complexes, which [91, 92] complicates the translation of in vitro potency to in vivo efficacy.

Regarding the chromatin complexes, it must be considered that there is an interplay among epigenetic factors for controlling gene expression. It has been described that targeting histone modifiers, such as the most common inhibitors of histone deacetylases (vorinostat, sodium butyrate, or trichostatine A), can result in changes on methylation of histones [93] and also alter the binding of chromatin remodelling factors at the same *locus*. HDAC inhibition may also alter the CpG methylation of specific promoters by affecting the activity of DNMTs [94]. One of the reasons for this interaction is that epigenetic modifiers work in complexes with other chromatin-related proteins [87]. Thus, the intervention on one partner can induce unforeseen effects on the other partners of the complex. It is really important to consider such cross-talk for in vitro genetic studies or assays for compound screening because effectively targeting a specific epigenetic enzyme in in vitro situations may not be effective in the cellular context.

In addition, several actual epidrugs do not show full selectivity. In one hand, isoform selectivity is not possible for several epidrugs. Development of isoform-selective inhibitors will be very useful because it can help to understand the role of each isoform. For example, in the case of the DNMTs, it would be very useful therapy-wise to know which DNMT is involved in which cancer or stage: is DNMT1 the best target or rather DNMT3A or 3B? On the other hand, multiple epigenetic enzymes from the same complex can be targeted. As an example, HDAC inhibitors can also repress the histone methyltransferase JARID1 [93], even if a lot of efforts are dedicated to designing specific inhibitors [95–98]. The development of multitarget inhibitors is in part addressing the latter point. It is based on the development of dual inhibitors, i.e. with two active motives targeting different epigenetic protein within the same molecule, which advantageously ensure simultaneous effect in cells of active entities, shorten clinical development, and minimise resistance phenomena. Thus, it appears to be of major interest to study the impact of hybrid inhibitors to improve the therapeutic management of cancer [22, 99]. An interesting example is the dual targeting of DNMT and G9A that induced prolonged survival in AML, ALL, and DLBCL xenogeneic models [100].

Another important issue is histone vs. non-histone selectivity. Substrate selectivity of epigenetic enzymes has been taken into consideration for a long time and evidence exist that substrates of epigenetic enzymes are not limited to canonical chromatin proteins [68, 92]. For example, the catalytic activity of the HAT p300/CBP includes acetylation at lysine residues of histone H3 and H4 but also acetylation of the oncogene p53 [91, 101]. Similarly, the histone HMT SMYD2 shows a strong preference for p53 peptide over histones as substrates for methylation [102]. Again, using in vitro assays based on nucleosomes substrates may not fully recapitulate the effect of the drug in a cellular context. In any case, this point should be considered when testing the off-targets of the compounds in cells.

This relates to the challenge of designing screening assays that takes into account the biological complexes of the enzyme and the structure of the chromatin. The expansion of High-Content Screening can address in part the challenge together with phenotypic screening that, as described above, has been important for the discovery of certain epigenetic drug. In parallel, the hit rates of the screening assays are low [103], suggesting that the design of focused chemical libraries for the epigenetic enzyme pockets that accommodate charged amino acid residues and hydrophilic molecules might increase the efficiency of lead discovery [104]. Fragment-based approaches are also explored in particular for protein targets that are poorly druggable by small-molecules. Cyclic peptides and stapled peptides are interesting chemical strategies to be further explored, together with the promising PROTAC strategy that induces the degradation of the protein targeted by the drug through E3 ligase recruitment and protease degradation [105, 106] Another challenge is the pharmacology of the compounds: the dose to be used (low doses have better effect for DNMT inhibitor s[107], or for how long to treat. The epigenetic reprogramming takes time, especially when the drug target are the ‘writers’ proteins: the epigenetic mark will be lost by passive demethylation or deacetylation [103, 108]. Does this mean that patients need to be treated for life? The recent clinical trials for the treatment of MLLr leukaemia with the inhibitor of DOT1L EPZ5676 are starting to address this question. Certainly, combination therapies, an epigenetic reprogramming followed by chemotherapy or immune-therapy, are promising strategies to eradicate cancer [109], but how to use the drugs in combination is to be optimised, e.g. first epigenetically reprogram and then treat with the immune or conventional therapy or administer both drugs simultaneously and so on.

It must be not forgotten that epigenetic factors are not only controlling gene expression in cis, that is to say, regulating directly the occupancy of the transcription factor machinery at specific *loci*. Epigenetic modifiers are also involved in the formation and maintenance of high-order chromatin structures regulating long-distance interactions [110]. The study of the role of ncRNAs in the regulation of the high-order chromatin structure is rapidly increasing and supporting the epigenetic regulation of genomic sequences in trans [111, 112].

From a clinical point of view, reprogramming the epigenetic landscape to combat chronic diseases requires a long-lasting effect. In this respect, inhibition of one epigenetic enzyme is not expected to result in sustained effects. Using epigenetic editing, many different epigenetic writers or erasers can be simultaneously targeted to a *locus* of interest to rewrite the epigenetic signature in such a way that the effects might be mitotically stable for many cell divisions [90, 113].

Although several on-going clinical trials exist on a wide range of tumoural and non-tumoural diseases, so far the use of epigenetic drugs in clinical practice is mostly limited to haematological malignancies [114, 115]. The potential of epigenetic drugs is extending to other pathologies, from infectious diseases to brain diseases, cardiovascular and metabolic disorders [5, 116]. This is very promising and we expect to observe interesting results in the following years. However, development of clinical trials requires the identification of biomarkers that can predict the drug response and avoid unnecessary side effects in patients with non-sensitive tumours. Both epigenetic mutations (i.e. promoter hypermethylation of tumour suppressor genes) and genetic mutations of epigenetic enzymes (loss or gain of function) can be used as predictors of response to chemotherapy in several cancer types [117]. For example, the epigenetic-associated silencing of MGMT is used as biomarker to predict the response to temozolomide treatment in glioblastoma patients [118].

## Conclusions

Current developments shed light on the importance of combining epidrugs (and also epidrugs with non-epigenetic therapeutic agents) that help to define improved policies for cancer treatment and non-cancer diseases. Validation of (epigenetics) biomarkers will assist in diagnosis, prediction to drug response and eventually identifying the responsive patients. All in all, researchers from various disciplines (chemists, biologists, clinicians) need to work together to optimise the drug engineering, read-out assays and clinical trial design.

## Data Availability

Not applicable

## Abbreviations

5-azaC: 5-Azacytidine
5-aza-dC: 5-Aza-2′-deoxycytidine
AML: Acute myeloid leukaemia
ATRA: All-trans retinoic acid
BET: Bromo and extra terminal
CMML: Chronic myelomonocytic leukaemia
CTCL: Cutaneous T cell lymphoma
DNMTs: DNA methyltransferases
DMSO: Dimethyl sulfoxide
HDACs: Histone deacetylases
JmjC: Jumonji C
KDMs: Histone lysine demethylases
KMTs: Histone methyltransferases
MAOs: Monoamine oxidases
MDS: Myelodysplastic syndrome
MLL: Mixed-lineage leukaemia
NMC: NUT midline carcinoma
ncRNAs: Non-coding RNAs
PTCL: Peripheral T cell lymphoma
SAHA: Suberoylanilide hydroxamic acid
SAM: S-Adenosyl methionine
VPA: Valproic acid
